# Health workforce issues and the Global Fund to fight AIDS, Tuberculosis and Malaria: an analytical review

**DOI:** 10.1186/1478-4491-4-23

**Published:** 2006-08-24

**Authors:** Sigrid Dräger, Gulin Gedik, Mario R Dal Poz

**Affiliations:** 1 Department of Human Resources; Evidence and Information for Policy; World Health Organization, Geneva, Switzerland

## Abstract

Recent studies have shown evidence of a direct and positive causal link between the number of health workers and health outcomes. Several studies have identified an adequate health workforce as one of the key ingredients to achieving improved health outcomes. Global health initiatives are faced with human resources issues as a major, system-wide constraint. This article explores how the Global Fund addresses the challenges of a health workforce bottleneck to the successful implementation of priority disease programmes.

Possibilities for investment in human resources in the Global Fund's policy documents and guidelines are reviewed. This is followed by an in-depth study of 35 Global Fund proposals from five African countries: Ethiopia, Ghana, Kenya, Malawi and Tanzania. The discussion presents specific human resources interventions that can be found in proposals. Finally, the comments on human resources interventions in the Global Fund's Technical Review Panel and the budget allocation for human resources for health were examined.

Policy documents and guidelines of the Global Fund foster taking account of human resources constraints in recipient countries and interventions to address them. However, the review of actual proposals clearly shows that countries do not often take advantage of their opportunities and focus mainly on short-term, in-service training in their human resources components.

The comments of the Technical Review Panel on proposed health system-strengthening interventions reveal a struggle between the Global Fund's goal to fight the three targeted diseases, on the one hand, and the need to strengthen health systems as a prerequisite for success, on the other. In realizing the opportunities the Global Fund provides for human resources interventions, countries should go beyond short-term objectives and link their activities to a long-term development of their human resources for health.

## Background

In the midst of accelerating advances in medicine and health technologies and a growing number of effective and affordable interventions, several low-income countries have experienced a decline in their health outcomes. Rates for child mortality are increasing and life expectancy is decreasing. There is a consensus emerging that one of the key ingredients to achieving improved health outcomes is stronger health systems, including an adequate health workforce [1]. Recent studies also show evidence of a direct and positive causal link between numbers of health workers and health outcomes [2,3].

The recently launched *World health report 2006 *suggests a minimum worker density threshold of 2.3 workers (doctors, nurses and midwives) per 1000 population necessary to achieve the United Nations Millennium Development Goals (MDGs) for health. WHO estimates a shortage of 2.4 million doctors, nurses and midwives worldwide, reaching a total of 4.3 million if all the workers required to manage and support their activities are included. The cost of training enough people to meet the shortfall by 2015 is on the order of USD 92 billion, and thereafter a minimum of USD 39 billion per year is required to pay their salaries [4].

The health workforce gap is one of the major bottlenecks to the success of global health initiatives. For example, two studies found that health workforce constraints were the key issue in successful implementation of the Global Alliance for Vaccines & Immunization (GAVI) programme [5]. Findings of these studies have implications for the Global Fund, as the Global Fund's approach has many similarities with GAVI. In light of the GAVI experience, one author suggests that: "staff shortages, poorly motivated staff, and the lack of resources for carrying out routine supervisory visits will inevitably be an obstacle to strengthening ... services for the three diseases" [6].

This review explores how the Global Fund addresses these challenges. It is outlined along the following key questions:

• Do the Global Fund policy and its guidelines offer any opportunity for investments in human resources for health?

• To what extent do countries make use of these opportunities?

This review first explores possibilities for investment in human resources in the Global Fund's policy documents and guidelines.

Next, five African countries were selected to study how the Global Fund proposals addressed human resources constraints. Thirty-five proposals from Ethiopia, Ghana, Kenya, Malawi and Tanzania that were submitted and approved for funding in the first five Rounds were reviewed in detail.

Finally, the comments on human resources interventions in the Global Fund's Technical Review Panel and the budget allocation for human resources for health were examined.

## The Global Fund

### Purpose and processes of the Global Fund

Since 2001, the Global Fund to Fight AIDS, Tuberculosis and Malaria has played an important part in the world's commitment to improve health. The Global Fund was created fundamentally as a financing agency. Its purpose is:

"to attract, manage and disburse additional resources through a new public-private partnership that will make a sustainable and significant contribution to the reduction of infections, illness and death, thereby mitigating the impact caused by HIV/AIDS, tuberculosis and malaria in countries in need, and contributing to poverty reduction as part of the Millennium Development Goals" [7].

The scope of the Global Fund comprises a substantial increase in coverage of proven and effective interventions for both the prevention and treatment of the three diseases. Activities supported by the Fund may also include efforts to strengthen health systems and human resources capacity.

One principle of the Global Fund is to support the integration of proposals into existing national and international disease programmes and implementation strategies. National ownership based on the equal partnership of local private and public stakeholders is another principle. The Fund seeks the active participation of local representatives from civil society and of those directly affected by the three diseases. Grants are disbursed to and implemented by a local principal recipient chosen in a country-led process. The flow of funds is closely linked to performance and progress, measured according to clearly defined monitoring and evaluation procedures.

Countries interested in receiving support from the Fund establish a local public-private partnership, the Country Coordinating Mechanism (CCM). The CCM prepares the proposals based on local needs and gaps in national programmes. To ensure an independent transparent process for the approval of proposals, the Global Fund relies on a Technical Review Panel (TRP) appointed by the board. International health and development experts assess the grant proposals and refer their recommendations to the board.

### Research on the Global Fund's impact on health systems

Even though the Global Fund attracts the interest of external researchers, few studies take a health systems perspective into account. An effort to study the Global Fund's long-term effects on health care systems is undertaken by the "Systemwide Effects of the Fund (SWEF) Research Network". SWEF seeks to understand how monies disbursed by the Global Fund and other significant funding agencies, such as the Multi-Country HIV/AIDS Programme (MAP) and the United States President's Emergency Plan for AIDS Relief (PEPFAR), affect the broader health care system in the recipient countries. SWEF has begun in-depth case studies in eight countries to study effects upon the policy environment, the public/private mix, human resources and pharmaceuticals [8].

The first SWEF observations allude to the fact that skills, motivation and distribution of health workers are likely to be affected by Global Fund projects [9]. Interim findings from Benin, Ethiopia and Malawi published in 2005 found that so far, human resources constraints have mainly been observed at the programme management level, which is probably due to the fact that implementation is still at a very early stage. At the programme management level it has also been noted that the employment of staff for the Global Fund on short-term contracts with salaries substantially higher than regular government employees has in some cases led to the exodus of technical staff from the ministry of health (MoH). At the health service delivery level, similar incidences have been observed with regard to health workers moving into higher-paid disease-specific positions. This could potentially weaken community-based services that are not related to one of the three target diseases [10].

One of the preliminary conclusions of the SWEF ongoing research is that health workforce planning must be strengthened in the context of scaling up activities [11] (O. Smith, personal communication, 2004).

Another observation from a Global Fund study highlights the strong linkages between macroeconomic policies and human resources for health (V.M. Nantulya, personal communication, 2005). The limitations introduced on public expenditure/GDP ratio as part of countries' fiscal policies in complying with International Monetary Fund requirements may result in ceilings on the recruitment of public servants and therefore on the recruitment of health workers. These restrictions may affect the feasibility, success and sustainability of projects of the Global Fund to scale up interventions in the fight against HIV, tuberculosis and malaria.

## The Global Fund's guidelines on human resources

### Health systems requirements

Since Round 1, proposals have been required to take into account the "institutional and absorptive capacity" of the country. Since then, the Global Fund has entered a kind of tightrope walk by focusing on its clearly defined goal to fight the three targeted diseases, but at the same time recognizing that adequate capacity of the health system is a prerequisite for any successful intervention.

As the guidelines have developed over the six Rounds, the necessity of functioning health systems and sufficient provision of human resources have been increasingly emphasized in the context of the required absorptive capacity of the recipient country.

Over the past Rounds, possibilities to support the health system through Global Fund resources are extended, but always require a link to the targeted diseases. According to guidelines for Round 1, a proposal "*may *include interventions to improve national capacity associated with the delivery and monitoring of programmes but should not have capacity building as its main focus." Since Round 4 the linkage of the proposed interventions to health systems strengthening has partly lost its optional character: "proposals *should *include the broader cross-cutting aspects of systems development that benefit the fight against AIDS, tuberculosis and/or malaria, and *should *describe how the proposal will have positive system-wide effects." Examples include human capacity development and infrastructure development.

Round 4 explicitly permitted an "integrated" proposal addressing a comprehensive response to the three diseases that focuses on system-wide effects. The guidelines for Round 5 also provide the opportunity for a "health system strengthening" proposal. In Round 6, however, the guidelines no longer allow separate health system proposals; activities for the strengthening of health systems now must be integrated within the disease-specific proposals.

### Integration with national plans and the potential for sustainability

Since Round 1, the guidelines have strongly encouraged links to existing national efforts to develop sustainable health systems and broader poverty reduction strategies. In order to demonstrate the potential for sustainability, countries are asked to describe links between the component and broader development policies and programmes such as Poverty Reduction Strategies or Sector-Wide Approaches. Round 4 adds requirements concerning the integration of the proposal with broader efforts to reach the Millennium Development Goals (MDGs) and other international initiatives.

Concerning the integration of proposals into public expenditure frameworks, guidelines gradually require more specific information. Round 5 guidelines ask to demonstrate "the ability to service recurrent expenditures" and in Round 6 countries need to describe "any relevant constraints e.g. budget or public sector spending ceilings".

With regard to linking the proposals to national health planning, the guidelines for proposals do ask for disease-specific national plans, whereas integration into an overall health development plan is not obligatory. However, the recent guidelines ask that proposals report if the current systems will be able to achieve and sustain any planned scale-up of intervention and what constraints exist. If health system constraints exist and adequate means to fully address these constraints are presently not available, applicants are "*encouraged *to include funding in respect of such activities" to strengthen the health system.

Integration with national plans is an important aspect of the potential for sustainability that characterizes a successful proposal, according to criteria laid down for the Technical Review Panel of the Global Fund. High-level political involvement and commitment with respect to the allocation of national resources is another aspect of sustainability.

In general, resources sought from the Global Fund should build on or scale up existing efforts and fill existing gaps in national budgets and funding from international donors. Complementarity and supplementation of the planned interventions must be demonstrated. It is a Global Fund policy that its disbursements should not replace existing national and international resources.

### Restrictions and special requirements for human resources for health

Since Round 2, the budget section of the proposal form has included a section on human resources that states: "In cases where human resources (HR) is an important share of the budget, explain to what extent HR spending will strengthen health systems capacity at the patient/target population level, and how these salaries will be sustained after the proposal period is over."

This very specific request for sustainability of salaries (which cannot be found for any other activities financed by the Global Fund) is closely linked to macroeconomic policies. It requires an intersectoral approach ensuring government commitment.

In addition, proposals are asked to demonstrate a direct link between spending on the health workforce and effects on the patient/target population. Studies have shown this link at the macro level [2,3] demonstrating a correlation between health outcome indicators (i.e. child mortality) and the density of the health workforce. However, it is quite a different challenge to prove such a direct connection for specific interventions to invest in human resources for health that are described in Global Fund proposals. One of the main obstacles is certainly the long time lag, which is typical for many investments for the health workforce, such as training of health professionals.

In the past, the Global Fund guidelines did not clearly define how the link between the health systems-strengthening activities and the disease-oriented goals should be made. It is only in Round 6 that the guidelines aim to provide a common framework for the required linkages between disease-specific and health systems interventions.

In summary, though, it is noteworthy that the Global Fund guidelines and proposal forms are in principle flexible and open to funding health workforce interventions. No explicit restrictions are evident for both short-term and long-term investments in human resources for health.

### Monitoring and evaluation

Since Round 4 the Global Fund has also provided a Monitoring and Evaluation Toolkit that was designed to support countries in measuring and reporting programme progress. The initial health workforce indicators suggested were the number of people trained and the number of health personnel with adequate supervision and motivation. The revised second edition, from January 2006, offers a wider range of indicators, such as annual output of trained health workers, number of health facilities fully staffed according to national standards or even patient satisfaction. Still, there is a heavy focus on indicators related to training.

Suggested indicators are far from comprehensive, and other indicators to follow up the impact of health workforce interventions may be used. This allows for country-specific indicators.

## Human resources in Global Fund proposals

### Involvement of health workforce stakeholders

In more than half of the reviewed proposals, some members of the CCM can be identified as stakeholders for human resources issues. This includes mainly representatives of academic institutions, professional associations or the ministry of education. For the majority of the members, however, and particularly with regard to the ministry of health, it is not possible to determine whether a person is an expert in one of the diseases or in health systems or in another category altogether: the composition of CCMs cannot be traced. Nevertheless, it cannot be denied that the composition of the CCMs and the choice of experts to write the proposals influences the content and perspective of the proposed projects.

To look further into this aspect, the list of documents attached to the proposals was examined. Here a noteworthy number of documents related to national disease strategies was included in most cases, but rarely any strategic paper on health systems development or human resource development.

The report of the Technical Review Panel on Round 5 proposals states: "The TRP is concerned that CCM composition has been built up based on the three diseases, so that many CCMs may lack the expertise to develop strong (health systems) proposals."

Guidelines on the CCMs have been changed several times, but the participation of cross-cutting experts has not yet entered the list of requirements for the CCM. Interestingly, for the Technical Review Panel of the Global Fund, a balanced composition of experts with either a disease-specific or health systems background is carefully monitored and reported [12,13].

### General health workforce concerns and the national context

The majority of proposals do take human resources issues into consideration. Out of the 35 reviewed proposals, 25 proposals across all five reviewed countries explicitly recognize human resources constraints. Nine out of 10 that did not tackle the issue are Round 1 and 2 proposals. This suggests a strong and increasing awareness of human resources as essential for the successful implementation of the programmes. Country examples may illustrate the sense of urgency:

• Tanzania: "The human resource shortage in the health sector is a major concern in Tanzania." (Round 4 Proposal HIV/AIDS)

• Malawi: "Presently, human resources constraints limit the capacity to implement available malaria control measures"...."Past experience indicates that development efforts failed because of lack of attention to human and institutional capabilities" (Round 2 Proposal Malaria); and even stronger formulations in Round 5: "The health system's civil service suffers from one of the worse staffing shortages in Africa creating a near breakdown in capacity to deliver basic level of health care especially in rural areas. Unless the crisis is resolved, Malawi will not have a sustainable public health system or capacity to expand treatment programs such as ART." (Round 5 Proposal HIV/AIDS and HSS (health system strengthening))

• Ethiopia: "The health system is affected by serious shortage of qualified human resources coupled with high turnover of trained staff." (Round 4 Proposal HIV/AIDS)

• Ghana: "Retention of service providers, their motivation and moral boosting will be crucial for the reduction in the disease burden of the three diseases." (Round 1 Proposal HIV/AIDS)

• Kenya: "Human resource capacity strengthening is key to all the efforts and will subsequently improve not only the delivery of TB services but also the overall functioning of the general health system in supported areas." (Round 4 Proposal Tuberculosis)

In accordance with the Global Fund's guidelines, nearly all proposals describe their links with national disease programmes and how the proposed interventions will fill identified gaps. However, broader development plans for the strengthening of health care systems are rarely included. Only seven out of 35 proposals mention a human resources development plan or a similar strategy.

Looking at the development over all five Rounds, the importance of a human resources development plan is emphasized more clearly in the later Rounds. In Ghana for example, the Round 5 proposal for tuberculosis requests the recruitment of three experts to work in the health-workforce development department of the MoH to develop a TB-specific human resources plan.

In some cases, over the short timespan of the 5 rounds, progress within a country can be observed. Kenya's Round 2 tuberculosis proposal states that the National Leprosy and Tuberculosis Programme is participating in the TBCTA (Tuberculosis Coalition for Technical Assistance) training initiative that is exploring ways to develop multi-year health workforce development plans. The Round 4 HIV/AIDS proposal expresses a specific intention to develop a human resources plan.

In Malawi the development becomes even more visible. Whereas the Round 1 HIV/AIDS proposal mentions that health delivery systems must be developed through incorporating human resources development, the Round 5 proposal on HIV/AIDS and health system strengthening (HSS) can draw on a fully developed and costed Emergency Human Resource Plan that is already partly funded by other donors; consequently the country asks the Global Fund to fill funding gaps.

### Specific human resources interventions in the proposals

#### Training

Training is one of the main topics in proposals. More than 90 % of the 35 proposals reviewed propose activities in this respect. The main focus of the training is in-service training for all types of health care workers. There is training of shopkeepers in good drug dispensary practices; training for prison services health staff; epidemiology courses for staff in public health institutions; data management and reporting skills for the monitoring and evaluation of the programme; and international training in public health for senior programme staff. The Round 2 HIV/TB proposal for mainland Tanzania, for example, states: "Twenty types of interrelated training activities for more than 10 000 participants from community groups/sites to national facility levels will be conducted."

Noticeable are the numerous training programmes for community health workers, lay counsellors, persons living with HIV/AIDS and other volunteers or peer educators who may support health care professionals on the front line. Ethiopia's Round 2 malaria proposal, for example, is to train more than 4000 community health workers and nearly 35 000 mother coordinators at the community level. A large proportion of non-health workers are also included in training for peer education among teachers and teenagers, people in the workplace, mobile population, sex workers and men having sex with men.

Most proposals provide an elaborate training strategy, often with a snowballing system in which the first generation is trained with the assistance of local academic institutions or developing partners. However, none of the reviewed proposals link their plans for capacity development to a coordinated country training plan. In fact, such plans seem to be lacking. Furthermore, only about 20 % of the suggested training programmes include the assessment of staff training needs, an evaluation of on-the-job-impact of new skills or any other kind of follow-up of training activities. A good practice example is provided in the Round 5 HIV/AIDS proposal from Ghana. The provision of follow-up after training includes "using creative approaches such as skills application plans, transfer of learning from classroom to real work life, and the provision of job aids (fact sheets, checklists) and other learning/work guides".

#### Support of pre-service training institutions

Few interventions that support pre-service training institutions can be found in the proposals. Updating curricula is a frequent activity but seems to be mainly an isolated exercise for the special Global Fund training programmes that are often limited to in-service training. Chances to revise curricula for all national training institutions are rarely taken; clearly this is also a matter of competence and involvement of stakeholders. One good practice example can be found in Kenya, where the Round 2 proposal on tuberculosis states that the need to incorporate TB control in the teaching curriculum of doctors and nurses is recognized and the National Leprosy and Tuberculosis Programme "has made this recommendation to the institutions." In Round 4 the inclusion of TB/HIV in curricula for middle- and high-level health institutions is a specific activity of the proposal with "number of schools using revised curricula" as an indicator for success.

Only one of the reviewed countries suggests a strategy that addresses the very root of the lack of human resources in supporting training institutions. Already in the Round 1 proposal on HIV/AIDS Malawi planned an expanded increase of medical students at its college of medicine and the establishment of a faculty of pharmacy. In the Round 5 HSS component, a strong rationale for the inclusion of pre-service training in the proposal is provided. It is argued that the general lack of labour supply and the critical need of HR managers plus insufficient career opportunities are the main reasons for low staff morale and migration among health workers and that this can be effectively addressed only through strengthening of training institutions. The four main training institutions play a pivotal role in this approach and have already provided "detailed plans for expansion and made output commitments to the MoH".

#### Recruitment

The analysis of recruitments planned in the proposals shows a common pattern. In 80 % of the proposals some recruitment is planned for the programme management level. New posts are created for administrators, accountants, procurement and logistics experts or similar positions. Staff are often hired only for the project time and may include external consultants, or secondments from the ministry of health. Few of these positions have a long-term perspective, although they might be supported by other donors after the proposal period is over, as suggested in one of the proposals.

On the other hand, greatly increased recruitment is planned for volunteer community health workers and volunteer staff for peer education, counselling and home-based care. In some cases, a small salary for the duration of the project is planned for these volunteers; in other cases non-monetary incentives or no remuneration is proposed. One proposal, for example, defines community health workers (CHW) as being recruited among community volunteers, retired school teachers, traditional birth attendants, etc. These CHW will not be given salaries but "funds will be made available to enable them to carry out their assigned activities" i.e. "enablers" such as free drugs, food packages, travel and transport allowances will be given. Many proposals do not provide detailed information in this matter and further research at the country level is necessary to analyse the use of volunteers and peer educators through Global Fund activities.

Apart from an increased pool of volunteers, a common strategy for the programme implementation level is also to obtain the necessary human resources from other partners, such as extended NGOs and faith-based organizations, that are expected to support their own staffing costs with the help of additional donor funding.

This review clearly shows that the number of core health workforce categories is rarely increased through Global Fund resources; countries were forced to look for more creative solutions to address human resources constraints. Ghana's Round 5 HIV/AIDS proposal puts it like this: "Scaling up national program entails increasing the number of workers but also extending the type of participants contributing to outcomes."

Only in the fourth round can the first example be found in which additional human resources are hired, such as for district TB clinics. The reluctance to recruit human resources for health is caused mainly by restrictions due to public sector freezes, which are mentioned several times in the proposals. An exceptional case of major resources (50% of the requested funds) planned to be used for recruitment and salaries of core health workers such as nurses and physicians is the Round 5 HSS proposal of Malawi. The country justifies its proposal, stating that for Malawi, the "nature of the health workforce crisis is such that the situation warrants measures that might not otherwise be considered as sustainable".

#### Motivation and retention strategies

About 60 % of the reviewed proposals consider motivation and retention strategies an important issue. In spite of some proposals that recognize the issue but do not provide any strategies to address it, an overview of the proposals gives a wide range of activities and some creative approaches. Monetary incentives in addition to salaries are a controversial topic and are supported by some proposals and not mentioned by others. Some remuneration strategies for volunteers who will not be paid a salary include travel and transport allowances, provision of motorbikes, and skills-building and training opportunities.

Performance based incentives and improved feedback and supervision systems are also suggested activities. The introduction of worker support meetings to help deal with burnout and stress is proposed in support of health care workers dealing with HIV/AIDS patients. One proposal plans to develop a national code of practice to combat stigma. Many health facilities ensure post-exposure prophylaxis and treatment for their staff.

In nearly all cases, only one or two single activities are suggested to address motivation issues, but no embedding strategy on motivation and performance is provided and the effectiveness of these activities is neither monitored nor evaluated. As a good practice example the Kenyan Round 4 tuberculosis proposal plans the implementation of a package of incentives to retain staff in hard-to-reach areas. This package includes a limited-stay policy and improved communication and training opportunities. Another comprehensive approach can be found in the Ghana Round 5 HIV/AIDS proposal, where motivation for lab personnel is addressed through a combination of training and capacity development of collaborative research. Again though, no measurement strategy is suggested to evaluate the effects on motivation and performance of health workers or retention rates.

#### Skill mix and regulation

A third of the reviewed proposals refer to skill mix as a regulation issue. Most of them tackle the creation of new cadres of volunteer and front-line workers such as lay youth counsellors and care supporters for home-based care. In one case, the formal government approval of a new category of dedicated counsellors as part of the civil service is sought as a Global Fund activity.

Some proposals include the adaptation of national standard guidelines and protocols to WHO guidelines, such as the development of guidelines for referral systems in their activities. One outstanding example plans the accreditation of all health services able to provide antiretroviral therapy, according to national guidelines with the support of Global Fund resources.

It is obvious, though, that skill mix and regulation of human resources for health is strongly affected by Global Fund activities, especially through the creation of numerous new types of front-line health workers. Long-term effects on health service delivery as well the role of the professional associations in the country coordinating mechanism in this process pose interesting questions for future research.

#### Sustainability

According to the Global Fund guidelines, proposals must tackle the issue of health workforce sustainability in cases where the health workforce accounts for an important share of the budget. Several proposals make a statement with regard to sustainability of salaries, but – not surprisingly – cannot provide settled agreements for the time after the proposal period is over. Vague statements may be found, such as "staffing costs will need to be absorbed into the MOHP or other service delivery organization budgets" (Malawi Round 1 HIV/AIDS) or "government should be able to absorb the additional staff" (Kenya Round 4 tuberculosis). The Round 5 HSS proposal by Malawi takes an innovative approach, stating that these human resources investments will "yield significant return with increment upgrading health systems productivity, accessibility and equity".

## Human resources in the Global Fund's review process of the proposals

The Global Fund's Technical Review Panel conducts its review based on the terms of reference laid down by the Global Fund [14]. General characteristics of successful proposals, such as soundness of approach and feasibility, are supplemented by the respective guidelines in force for that specific Round. No specific criteria can be found on health systems or human resources issues, apart from the advice that the potential for the sustainability of the approach should be outlined. Sustainability in this context refers to "the capacity to absorb increased resources such as through innovative approaches to overcoming human resource capacity constraints". This passage was introduced into the guidelines in Round 4 and can also be found in Round 5 and 6 guidelines. Nonetheless, the inability of proposal writers to provide more detailed information on how to ensure sustainability of salaries for human resources did not receive any negative feedback from the TRP.

In general the consideration of human resources issues and the integration of human resources components in the proposals is welcomed by the Technical Review Panel of the Global Fund. The TRP counts it as a strength, for example, that "major risks, especially weaknesses in the health system, including human resources" are named in a proposal and specific measures are taken to address them.

An inadequate consideration of human resources constraints has repeatedly led to critical comments of the TRP and doubts about the successful achievement of the project goals. A lack of discussion on how ambitious scaling-up targets can be achieved, given the countries' present human resources constraints, is one of the reasons that has led to a rejection of proposals.

In proposals that do address human resources issues, key weaknesses are a lack of a comprehensive situation analysis for the health workforce and a lack of overall health workforce development plans [15,16]. Interventions for the strengthening of the health workforce mainly address single issues without providing an embedding strategy for long-term success. Some activities, for example, aim at a better working environment and improved health worker performance, but no comprehensive motivation and retention strategies are provided along these lines. Numerous training activities are suggested, but they are not part of a coordinated country training plan.

In addition, it often remains unclear how the proposed interventions for the health workforce will have positive effects on the patient/target population. Countries struggled to propose clear approaches for the monitoring and evaluation of the success of their health workforce interventions. This is also due to the poor standard of current human resources information systems (HRIS), although this issue has not been specifically addressed by the TRP.

Table 1 illustrates an overview of Round 4 and 5 TRP comments on human resources interventions:

**Table 1:** Round 4 and 5 comments of the Global Fund's Technical Review Panel on human resources for health

**Positive TRP comments**	**Negative TRP comments**
Proposal could make a significant contribution to the underlying structural difficulties preventing adequate response to AIDS, Tb and malaria Link to specific disease well articulated Standardization of training curricula Involvement of training institution Involvement of private sector National code of practice for health workers being developed to reduce stigma Key role of non-health personnel acknowledged	Insufficient assessment and planning of HR issues Limited human resources available – capacity insufficient to achieve goals Insufficient situational analysis in terms of human resources Training strategies lack an accurate description of partners/providers Unclear number of trainees and costs Inadequately addressing motivation issues

Source: [15, 16]

### Human resources in integrated and health system proposals

In Round 4, the guidelines introduce a specific option to address cross-cutting health systems issues, including human resources constraints, in an integrated proposal. This option, however, was not widely used by countries. There were only six integrated proposals out of 171 proposals in Round 4 and they were also less successful than the average in the Technical Review Panel. None of them was recommended for funding, although four may be resubmitted in subsequent rounds.

One of the integrated proposals from Uganda aims specifically at strengthening human resource development. Expanding the capacity of training institutions, in-service training, an incentive system for hardship areas and strengthened human resources management at all levels were the main features of this outstanding proposal. The TRP classified this proposal as not recommended for funding, reasoning: "it appeared questionable to us that the GFATM is asked to fund a relatively mid-long-term multi-sectoral programme, rather than focusing on gaps that urgently need to be addressed to implement the HIV, malaria and TB programmes that are ongoing... with immediate impact on the delivery of services." Here, the Global Fund's goal for a direct impact in fighting the three targeted diseases clearly outweighed the necessity for long-term investments.

For Round 5, the Global Fund decided to invite specific health system strengthening (HSS) components and countries responded more readily: 30 out of the 202 components reviewed in Round 5 were HSS proposals. Again, their success rate was well below the average: only three proposals (10%) are recommended for funding, compared to a 31 % overall success rate. With regard to budgets, this translates into the approval of USD 43 million for HSS proposals out of a total budget of USD 726 million in Round 5.

**Figure 1:**
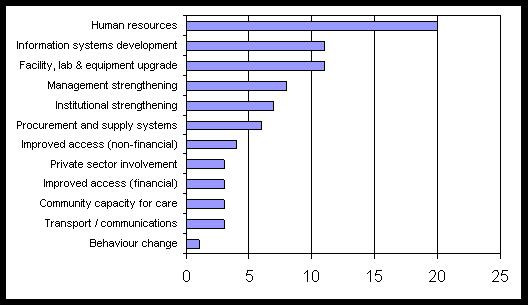
**The distribution of health systems' interventions in 30 proposals on health systems strengthening in Round 5**. Source: [17].

A review of the content of the 30 HSS components shows that human resources is by far the most commonly tackled issue (20 HSS proposals), followed by interventions for the development of health information systems. Specific improvement of the human resources information system cannot be found as a stand-alone intervention in any of the HSS proposals, but is usually part of the overall strengthening of the health information system. An overview of proposed interventions for strengthening health systems can be found in Figure 1 and Table 2.

**Table 2:** Activities for HRH development in Round 5 proposals (in 20 proposals that include HRH development)

**Activity for HRH development in Round 5 proposals**	**Number of proposals**
Training formal sector HRH	16
Incentives	12
Recruitment (all cadres)	6
Recruitment of expats	1
Workforce planning, data collection	2
Training "local people"/CHWs	2
Other HRH development activities	2

Source: [17]

Comments of the Technical Review Panel in Round 5 show quite positive reactions to human resource interventions planned in the HSS proposal. Two of the three successful proposals have a major human resources component. The HSS component from Rwanda assigns 80% of requested funds to human resources and training, the HSS component from Malawi even more than 90% of the budget. Commenting on the HSS proposal from Malawi the TRP reflects that although the human resources constraints were recognized as crucial in Round 1, "at that time the Global Fund was not keen to fund health system components", indicating that the approach of the Panel has undergone some change since the early Rounds.

In their report on Round 5 proposals, the TRP found that the invitation of separate HSS proposals was given "insufficient consideration". Applicants were unsure about the precise scope of HSS proposals and "were not given any specific guidance on what an effective linkage between HSS and a disease component should or could look like". This resulted in a high failure rate of Round 5 HSS proposals due to objectives that were too vague and ambitious and a contrived and superficial linkage between HSS activities and the specific diseases.

The Technical Review Panel summarizes that the poor quality of these proposals "reflects a confusion in the GFTM as to the precise mandate of the Fund in relation to HSS proposals." The Panel stated a need to refine the Fund's mandate in relation to HSS proposals and raised the question whether to retain HSS proposals as a separate category, especially as the proposal forms had been found "largely unsuitable for the submission of HSS proposals" [16].

Consequently, the option of separate HSS proposals was abolished for Round 6; health system interventions must now be completely integrated into the specific disease component section.

## Budget allocations for human resources

**Figure 2:**
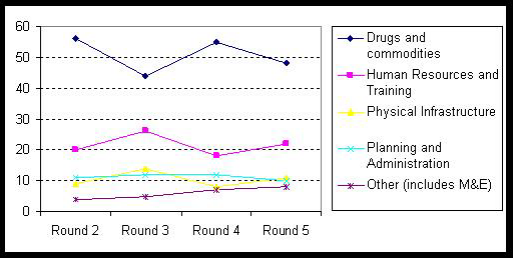
**Distribution of grants per Round by expenditure targets in % (provides summary of amounts for all Rounds to date)**. Source: [19, 20].

The Global Fund's reports on the distribution of grants show that 55% of the resources committed so far have been assigned to sub-Saharan Africa; 58% of the total funding is allocated to HIV/AIDS. Looking at the budget proportions per expenditure targets reveals a decrease in investment in human resources for health and training in Round 4. A recent study of all malaria components over all four Rounds also reveals a decline in allocation to human resources and training towards Round 4 [18]. Then again, in Round 5 there is a slight increase in the budget allocation towards human resources for health and training (Figure 2).

Here it must be taken into consideration that the total amount approved for grants in Round 3 was lower than in the other rounds (Table 3).

**Table 3:** Overview of approved proposals

**Round**	**Countries**	**Approved amount, USD**
Round 1	35	1 301 787 563
Round 2	73	1 506 465 345
Round 3	63	644 322 679
Round 4	65	1 013 856 978
Round 5	50	779 013 319
**All rounds**	**131**	**5 245 445 884**

Source: [20]

This evolution of the budget allocation for human resources, however, is not readily accessible for interpretation, especially as the definitions of the budget categories "human resources" and "training" have been changed over time.

On the one hand, human resources costs are sometimes hidden within other components: training on the application of a new drug might be included in the category "Drugs", for example. On the other hand, the health workforce budget might include resources for workshops and meetings, for example, without having a specified proportion for human resources within these lump sums. An initial study of the malaria proposals in 14 African countries up to and including Round 3 that was conducted by the Roll Back Malaria Department at WHO Geneva found that of the 25% that were declared as the human resources and training proportion of total funds, only around 10% could still be defined as human resources and training costs after a more detailed analysis.

In addition it is noteworthy that in the 35 African proposals reviewed, the proportion of funds assigned to the budget categories "human resources" and "training" summed up to only 16% of funds over five Rounds (human resources 6.6%, training 9.2%). This is considerably lower than the average of 22% for the distribution of the total sum of global funds as shown in Figure 2. This finding is surprising, because all five of the reviewed African countries are frequently identified with issues related to human resources constraints.

## Conclusion

The Global Fund clearly provides the opportunity to invest in health systems strengthening and human resources. Policy documents acknowledge that health system capacities and human resources in particular are a necessary prerequisite for the success of interventions that aim to fight the three targeted disease. Changes over time in the Global Fund's guidelines and proposal forms reflect ever more attention to the strengthening of health systems. However, there has been a struggle to accommodate health systems strengthening with the objectives of the Global Fund and its administrative guidelines. The change made in Round 6 guidelines to abandon the possibility for separate health systems proposals can be interpreted as a consequence of this dilemma.

In general, most countries do not sufficiently use the possibilities that the Global Fund provides for health system strengthening and human resources interventions, even though a great majority of proposals recognize human resources constraints as a key to success of future interventions. Most proposals include some activities to address human resources constraints. The most frequent activity is training, focused mainly on short-term, in-service training. Support for pre-service training and training institutions is rare and hardly any long-term strategies are proposed to address the lack of adequately trained personnel.

Recruitment plans are also frequently included in the Global Fund proposals. In most cases, however, this is limited to a small number of staff at the programme management level rather than addressing the shortages at the service delivery level.

This absence may also be due to the Global Fund's specific requirement to demonstrate the sustainability of salaries financed by the Global Fund after the proposal period is over. In the long run this will make it inevitable to address macroeconomic issues, particularly the restrictions imposed on the public sector by the hiring freeze.

The comments of the Technical Review Panel on proposed health system-strengthening interventions reveal a struggle between the Global Fund's goal to fight the three targeted diseases, on the one hand, and the need to strengthen health systems as a prerequisite for success, on the other. Unfortunately, in the past the TRP has tended to favour the Global Fund's disease-related objectives over cross-cutting issues.

In realizing the opportunities the Global Fund provides for human resources interventions, countries should go beyond short-term objectives and link their activities to a long-term development of their human resources for health. The existence of a human resources development plan, a clear identification of currents gaps and a linkage between human resources and coverage for the targeted disease have been favourite ingredients in proposals requesting funds for human resources. If human resource development plans are not presently available, the framework of the Global Fund permits support to technical assistance to lay those foundations.

One step that can be taken at the country level to take advantage of the opportunities the Global Fund provides for strengthening the health workforce is to ensure the involvement of human resources stakeholders and experts in both the CCM and the proposal writing groups.

As a preliminary observation on the development of proposals from Round 1 to 5 it can be said that the activities of the Global Fund have revealed and partly exacerbated some long-standing health system weaknesses, especially with regard to human resources. In order to successfully tap into the Global Fund's resources, countries are forced to provide needs-assessment and clear development plans for their human resources for health. Proposals of the five countries reviewed here clearly show some positive developments towards more comprehensive approaches to address human resources constraints. However countries need more encouragement and support to back up their human resources for health through the framework of the Global Fund.

## Competing interests

The author(s) declare that they have no competing interests.

## Authors' contributions

All authors contributed to the design and writing of this paper, and read and approved the final manuscript.
